# Supplementary material for: Intraoperative blood loss may be associated with myocardial injury after non-cardiac surgery
Source: PLoS One. 2021 Feb 24;16(2):e0241114. doi: 10.1371/journal.pone.0241114 (PMC7904206; doi:10.1371/journal.pone.0241114)
Supplement: S1 Table — (DOCX) [file pone.0241114.s001.docx]

**S1 Table.** Sensitivity Analysis of the Observed Association between Significant Bleeding and Myocardial Injury after Noncardiac Surgery

|  | OR (95% CI) | P-value |
| --- | --- | --- |
| Patients with heart failure (n=327) | 4.63 (2.42-9.05) | <0.001 |
| Patients with chronic kidney disease (n=1012) | 1.17 (0.86-1.59) | <0.001 |
| Surgeries after 2017 with the threshold of  hemoglobin level < 7 g/dL for intraoperative transfusion | 2.20 (1.94-2.50) | <0.001 |

OR, odds ratio; CI, confidence interval; RBC, red blood cell
