# Supplementary material for: Intraoperative blood loss may be associated with myocardial injury after non-cardiac surgery
Source: PLoS One. 2021 Feb 24;16(2):e0241114. doi: 10.1371/journal.pone.0241114 (PMC7904206; doi:10.1371/journal.pone.0241114)
Supplement: S2 Table — (DOCX) [file pone.0241114.s002.docx]

**S2 Table.** Sensitivity Analysis of the Effect of an Unmeasured Confounder on Odds Ratio of Significant Bleeding for Myocardial Injury after Noncardiac Surgery

|  |  | **OR*_ZY_*_\|_*_X_*** | | | | | |
| --- | --- | --- | --- | --- | --- | --- | --- |
|  |  | **1.5** | **2** | **2.5** | **3** | **3.5** | **4** |
| OR_zx_ | 0.3 | 2.15 (1.92-2.41) | 2.40 (2.14-2.70) | 2.68 (2.38-3.01) | 3.01 (2.67-3.40) | 3.32 (2.94-3.75) | 3.60 (3.17-4.08) |
|  | 0.4 | 2.05 (1.83-2.29) | 2.26 (2.02-2.53) | 2.45 (2.18-2.75) | 2.67 (2.38-3.01) | 2.84 (2.52-3.20) | 3.08 (2.73-3.48) |
|  | 0.5 | 1.99 (1.78-2.22) | 2.14 (1.91-2.40) | 2.29 (2.04-2.57) | 2.46 (2.19-2.76) | 2.60 (2.31-2.92) | 2.72 (2.42-3.07) |
|  | 0.6 | 1.94 (1.74-2.16) | 2.05 (1.84-2.29) | 2.16 (1.93-2.41) | 2.28 (2.03-2.55) | 2.39 (2.13-2.68) | 2.52 (2.24-2.83) |
|  | 0.7 | 1.89 (1.69-2.11) | 1.98 (1.77-2.21) | 2.08 (1.86-2.32) | 2.16 (1.92-2.42) | 2.27 (2.02-2.55) | 2.30 (2.05-2.59) |

Prevalence of unmeasured confounder = 40%

Numbers represent HRs (including 95% CIs).

OR, odds ratio; HR, hazard ratio; X: dichotomous exposure measure, y dichotomous outcome measure, z : potential dichotomous confounder.

OR_ZX_ indicates the association (OR) between the unmeasured confounder and significant bleeding.

OR_ZY|X_ indicates the association (OR) between the unmeasured confounder and mortality conditional on exposure status
