# Supplementary material for: Intraoperative blood loss may be associated with myocardial injury after non-cardiac surgery
Source: PLoS One. 2021 Feb 24;16(2):e0241114. doi: 10.1371/journal.pone.0241114 (PMC7904206; doi:10.1371/journal.pone.0241114)
Supplement: S3 Table — (DOCX) [file pone.0241114.s003.docx]

**S3 Table.** Baseline Characteristics According to the Actual Hemoglobin Decrease and Intraoperative Transfusion

|  | **No hemoglobin decrease without transfusion** | **No hemoglobin decrease with transfusion** | **Hemoglobin decrease without transfusion**  **(N = 275)** | **Hemoglobin decrease with transfusion** |
| --- | --- | --- | --- | --- |
|  | **(N = 13416)** | **(N = 1937)** |  | **(N = 298)** |
| Male | 8133 (60.6) | 1254 (64.7) | 142 (51.6) | 166 (55.7) |
| Age | 61.7 (±13.5) | 61.2 (±13.2) | 57.0 (±15.6) | 54.8 (±11.2) |
| Preoperative anemia | 4949 (36.9) | 1170 (60.4) | 208 (75.6) | 268 (89.9) |
| Diabetes | 7890 (58.8) | 1329 (68.6) | 171 (62.2) | 248 (83.2) |
| Hypertension | 7290 (54.3) | 1111 (57.4) | 141 (51.3) | 172 (57.7) |
| Current smoking | 1362 (10.2) | 165 (8.5) | 32 (11.6) | 39 (13.1) |
| Current alcohol | 2798 (20.9) | 265 (13.7) | 51 (18.5) | 44 (14.8) |
| Chronic kidney disease | 680 (5.1) | 227 (11.7) | 43 (15.6) | 62 (20.8) |
| History of ischemic heart disease | 1913 (14.3) | 299 (15.4) | 30 (10.9) | 36 (12.1) |
| History of heart failure | 282 (2.1) | 40 (2.1) | 4 (1.5) | 1 (0.3) |
| History of stroke | 938 (7.0) | 124 (6.4) | 26 (9.5) | 11 (3.7) |
| History of arrhythmia | 882 (6.6) | 139 (7.2) | 11 (4.0) | 11 (3.7) |
| History of heart valve disease | 149 (1.1) | 16 (0.8) | 5 (1.8) | 0 |
| Active cancer | 7422 (55.3) | 950 (49.0) | 91 (33.1) | 71 (23.8) |
| Preoperative care |  |  |  |  |
| RBC transfusion | 608 (4.5) | 87 (4.5) | 36 (13.1) | 268 (89.9) |
| Intensive care unit | 424 (3.2) | 146 (7.5) | 52 (18.9) | 80 (26.8) |
| ECMO | 0 | 1 (0.0) | 0 | 0 |
| Continuous renal replacement therapy | 18 (0.1) | 19 (1.0) | 12 (4.4) | 23 (7.7) |
| Ventilator | 67 (0.5) | 23 (1.2) | 11 (4.0) | 22 (7.4) |
| Operative variables |  |  |  |  |
| ESC/ESA surgical high risk | 3997 (29.8) | 908 (46.9) | 124 (45.1) | 226 (75.8) |
| Emergency operation | 1521 (11.3) | 333 (17.2) | 98 (35.6) | 118 (39.6) |
| General anesthesia | 13305 (99.2) | 1930 (99.6) | 272 (98.9) | 298 (100) |
| Operation duration, hours | 3.94 (±2.14) | 5.16 (±3.26) | 6.33 (±3.41) | 7.52 (±3.00) |
| Continuous infusion of inotropics | 4592 (34.2) | 891 (46.0) | 169 (61.5) | 218 (73.2) |

Data are presented as n (%) or mean (±standard deviation)

RBC, red blood cell; ECMO, extracorporeal membranous oxygenation; RAAS, renin-angiotensin-aldosterone system; ESC, European Society of Cardiology; ESA, European Society of Anaesthesiology
