# Supplementary material for: Intraoperative blood loss may be associated with myocardial injury after non-cardiac surgery
Source: PLoS One. 2021 Feb 24;16(2):e0241114. doi: 10.1371/journal.pone.0241114 (PMC7904206; doi:10.1371/journal.pone.0241114)
Supplement: S4 Table — (DOCX) [file pone.0241114.s004.docx]

**S4 Table.** Baseline Characteristics According to the Actual Hemoglobin Decrease without Regarding Intraoperative Transfusion

|  | **No hemoglobin decrease** | **Hemoglobin decrease** | ***P* Value** |
| --- | --- | --- | --- |
|  | **(N = 15353)** | **(N = 573)** |  |
| Male | 9387 (61.1) | 308 (53.8) | <0.001 |
| Age | 61.6 (±13.5) | 55.8 (±13.5) | <0.001 |
| Preoperative anemia | 6119 (39.9) | 476 (83.1) | <0.001 |
| Diabetes | 9219 (60.0) | 419 (73.1) | <0.001 |
| Hypertension | 8401 (54.7) | 313 (54.6) | 0.99 |
| Current smoking | 1527 (9.9) | 71 (12.1) | 0.07 |
| Current alcohol | 3063 (20.0) | 95 (16.6) | 0.05 |
| Chronic kidney disease | 907 (5.9) | 105 (18.3) | <0.001 |
| History of ischemic heart disease | 2212 (14.4) | 66 (11.5) | 0.06 |
| History of heart failure | 322 (2.1) | 5 (0.9) | 0.06 |
| History of stroke | 1062 (6.9) | 37 (6.5) | 0.73 |
| History of arrhythmia | 1021 (6.7) | 22 (3.8) | 0.01 |
| History of heart valve disease | 165 (1.1) | 5 (0.9) | 0.8 |
| Active cancer | 8372 (54.5) | 162 (28.3) | <0.001 |
| Preoperative care |  |  |  |
| RBC transfusion | 695 (4.5) | 64 (11.2) | <0.001 |
| Intensive care unit | 570 (3.7) | 132 (23.0) | <0.001 |
| ECMO | 1 (0.0) | 0 | >0.99 |
| Continuous renal replacement therapy | 37 (0.2) | 35 (6.1) | <0.001 |
| Ventilator | 90 (0.6) | 33 (5.8) | <0.001 |
| Operative variables |  |  |  |
| ESC/ESA surgical high risk | 4905 (31.9) | 350 (61.1) | <0.001 |
| Emergency operation | 1854 (12.1) | 216 (37.7) | <0.001 |
| General anesthesia | 15235 (99.2) | 570 (99.5) | 0.68 |
| Operation duration, hours | 4.09 (±2.24) | 6.95 (±3.26) | <0.001 |
| Continuous infusion of inotropes | 5483 (35.7) | 387 (67.5) | <0.001 |
| Types of surgery |  |  |  |
| Vascular | 1090 (7.1) | 31 (5.4) |  |
| Orthopedic | 800 (5.2) | 28 (4.9) |  |
| Neuro | 3449 (22.5) | 36 (6.3) |  |
| Breast or Endo | 165 (1.1) | 5 (0.9) |  |
| Plastic or Otolaryngeal or Eye | 318 (2.1) | 15 (2.6) |  |
| Transplantation | 773 (5.0) | 295 (51.5) |  |
| Gynecology or Urology | 1062 (6.9) | 38 (6.6) |  |
| Gastrointestinal | 5176 (33.7) | 112 (19.5) |  |
| Noncardiac thoracic | 2494 (16.2) | 13 (2.3) |  |
| Others | 26 (0.2) | 0 |  |

Data are presented as n (%) or mean (±standard deviation)

RBC, red blood cell; ECMO, extracorporeal membranous oxygenation; RAAS, renin-angiotensin-aldosterone system; ESC, European Society of Cardiology; ESA, European Society of Anaesthesiology
