# Supplementary material for: Intraoperative blood loss may be associated with myocardial injury after non-cardiac surgery
Source: PLoS One. 2021 Feb 24;16(2):e0241114. doi: 10.1371/journal.pone.0241114 (PMC7904206; doi:10.1371/journal.pone.0241114)
Supplement: S5 Table — (DOCX) [file pone.0241114.s005.docx]

**S5 Table.** The Incidence of Myocardial Injury after Noncardiac Surgery and Mortality According to the Actual Hemoglobin Decrease without Regarding Intraoperative Transfusion

|  | **No hemoglobin decrease**  **(N = 15353)** | **Hemoglobin decrease**  **(N = 573)** | **Unadjusted OR/HR (95% CI)** | ***p* value** | **Adjusted OR/HR (95% CI)** | ***p* value** |
| --- | --- | --- | --- | --- | --- | --- |
| MINS | 2749 (17.9) | 334 (58.3) | 6.41 (5.40-7.61) | < 0.001 | 3.28 (2.70-4.00) | < 0.001 |
| 30-day mortality | 234 (1.5) | 51 (8.9) | 6.11 (4.52-8.27) | < 0.001 | 2.52 (1.75-3.63) | < 0.001 |
| Cardiovascular | 67 (0.4) | 11 (1.9) | 4.58 (2.42-8.66) | < 0.001 | 2.01 (0.94-4.32) | 0.07 |
| Noncardiovascular | 167 (1.1) | 40 (7.0) | 6.73 (4.76-9.50) | < 0.001 | 2.73 (1.80-4.13) | < 0.001 |
