# Supplementary material for: Intraoperative blood loss may be associated with myocardial injury after non-cardiac surgery
Source: PLoS One. 2021 Feb 24;16(2):e0241114. doi: 10.1371/journal.pone.0241114 (PMC7904206; doi:10.1371/journal.pone.0241114)
Supplement: S6 Table — (DOCX) [file pone.0241114.s006.docx]

**S6 Table.** Sensitivity Analysis of the Effect of an Unmeasured Confounder on Odds Ratio of Hemoglobin Decrease for Myocardial Injury after Noncardiac Surgery

|  |  | **OR*_ZY_*_\|_*_X_*** | | | | | |
| --- | --- | --- | --- | --- | --- | --- | --- |
|  |  | **1.5** | **2** | **2.5** | **3** | **3.5** | **4** |
| OR_zx_ | 0.3 | 2.15 (1.92-2.41) | 2.44 (2.18-2.74) | 2.69 (2.39-3.03) | 2.98 (2.65-3.37) | 3.25 (2.87-3.67) | 3.63 (3.20-4.11) |
|  | 0.4 | 2.07 (1.85-2.31) | 2.26 (2.02-2.53) | 2.47 (2.20-2.78) | 2.68 (2.38-3.02) | 2.84 (2.52-3.21) | 3.08 (2.72-3.48) |
|  | 0.5 | 1.99 (1.78-2.23) | 2.13 (1.90-2.38) | 2.29 (2.04-2.57) | 2.44 (2.17-2.74) | 2.63 (2.34-2.96) | 2.72 (2.41-3.06) |
|  | 0.6 | 1.93 (1.73-2.16) | 2.04 (1.82-2.28) | 2.16 (1.93-2.42) | 2.27 (2.03-2.55) | 2.41 (2.15-2.71) | 2.51 (2.23-2.83) |
|  | 0.7 | 1.90 (1.70-2.12) | 1.97 (1.77-2.20) | 2.07 (1.85-2.32) | 2.14 (1.91-2.39) | 2.25 (2.01-2.53) | 2.34 (2.08-2.63) |

Prevalence of unmeasured confounder = 40%

Numbers represent HRs (including 95% CIs).

OR, odds ratio; HR, hazard ratio; X: dichotomous exposure measure, y dichotomous outcome measure, z : potential dichotomous confounder

OR_ZX_ indicates the association (OR) between the unmeasured confounder and hemoglobin decrease.

OR_ZY|X_ indicates the association (OR) between the unmeasured confounder and mortality conditional on exposure status.
