# Supplementary material for: Intraoperative blood loss may be associated with myocardial injury after non-cardiac surgery
Source: PLoS One. 2021 Feb 24;16(2):e0241114. doi: 10.1371/journal.pone.0241114 (PMC7904206; doi:10.1371/journal.pone.0241114)
Supplement: Attachment — Submitted filename: PONE-D-20-14264.docx [file pone.0241114.s007.docx]

Dear Dr Wen Chih-Wu

Thank you for inviting me to review this paper entitled “Intraoperative blood loss may be associated with myocardial injury after non-cardiac Surgery”. Strong points include the a priori registration of the cohort, the large dataset collected, and the novel and important research question. The authors have clearly gone to a great deal of trouble to extract an impressive cohort of patients and analyse it diligently. It has the potential to be publishable, but I think the following comments should be taken into consideration:

Major comments:

- Introduction: our current understanding of MINS is not, as the authors state, principally myocardial oxygen supply/demand imbalance (which more accurately describes Type 2 myocardial infarction according to the 4^th^ universal definition as published by the American College of Cardiology) but is pathophysiologically undefined. Exact causation has not been established but it is likely a heterogenous syndrome of cardiac myocyte necrosis secondary to ischemia, inflammation, immunological stress reaction (mostly through circulating cytokines such as TNF-alpha), and neuroendocrine dysregulation, and as such has a large number of potential clinical correlates. MINS is therefore more accurately a biomarker for increased postoperative morbidity and mortality from a range of sources.
- The authors have excluded patients with conditions that cause raised troponin elevation (such as renal impairment, heart failure, sepsis, atrial fibrillation, pulmonary embolism etc.) as these are regarded as artefactual troponin rises. This is can be a legitimate decision, but requires justification. These conditions are often co-morbid with MINS as they share a common pathology (inflammatory response to tissue trauma), so true MINS incidence may be missed. This could be screened for using a sensitivity analysis, as was done for renal impairment and heart failure in the original VISION paper (1), where the MINS concept was first popularised.
- There is a significant clinical and statistical difference in baseline characteristics between the bleeding and non-bleeding cohorts, with regard to anemia, alcohol use, chronic kidney disease, active cancer, being in ICU, continuous renal replacement therapy, ventilation, surgical risk, emergency operation status and requirement for continuous inotropic support. All of these affect the risk of MINS, and therefore confound the effect of bleeding on the incidence of MINS. This is a major limitation.
- The authors state that they have made an estimate of the effect of potential unmeasured confounders (presumably with a sensitivity analysis with a null variable although this is not explicitly stated). This technique requires more explanation, particularly in view of the very disparate and potentially powerfully confounding medical statuses of the two groups in the cohort. Sensitivity analysis is usually used in epidemiology to model the potential effect of a variable which is known or postulated from prior experience or research, but unmeasured in the study at hand. For example, “I think having X increases risk of MINS by OR 2, what effect would it have on my results if it 20% of my MINS group had X? What about 40%? Etc.” This enables you to make a statement such as “Even if X were present in one of my groups with prevalence up to 40%, it would not have changed the result”. How it was used in this study requires more explanation in the text. This technique does not compensate for the confounding caused by the major differences in medical status and operation type mentioned already.
- The authors analyse their data with two different definitions of significant blood loss (including and excluding whether transfusion was required). This is confusing. They could consider picking one definition of bleeding and analysing that as the primary outcome, and then if they wanted to explore the interaction of transfusion they could do that as a secondary analysis (perhaps using matched pairs controlled for hemoglobin drop or some similar technique). Alternatively, they could divide the groups into a two-by-two table (hemoglobin decrease/transfusion) and just make this the primary reported result (as they did in table s5), skipping the intermediate steps. This would be an important result, as transfusion has it’s own risks that may be associated with MINS, and disentangling it from the effect of the bleeding event itself, and anemia, is important.

Minor comments

- Line 135 – I assume “high risk” here refers to operative mortality risk >5% in table 3 of the ESC/ESA guidelines 2014 (2)? This could be made absolutely clear
- Line 65 and elsewhere – the authors give their main result as “**16.4% vs. 35.2%;** odds ratio, 1.80; 95% confidence interval, 1.61–2.00; *p* < 0.001” This should read “**35.2% vs 16.4%**; odds ratio 1.80; 95% confidence interval 1.61-2.00; p<0.001”. This is true for all the odds ratios in the text.
- While the authors tell us that these are patients having high risk surgery, they do not include a breakdown of the types of surgery performed in the table 1 (Baseline Characteristics). This is important enough to be in the main text, as it affects the applicability of these findings to our individual practice settings. In addition, some surgical populations (vascular surgery in particular) are at very high risk of MINS, and a different incidence between groups may confound the data. This should be moved up into the main text from the supplement (from table S1 into table 1). P values should also be provided.
- Line 130 – the exclusion criteria of factors that artefactually increase MINS should be moved up into the methods section for clarity.
- General – this dataset spans a relatively long period of time, beginning in 2010, during which our understanding of the hazards of transfusion has evolved and trigger levels of hemoglobin for transfusion have dropped from 10 to 7 g/dl. Were there changes to the author’s institutional policy or consensus in practice on transfusion triggers during this time? Did the authors consider a temporal analysis either on lowest hemoglobin before transfusion, or numbers of transfusions?
- Details of the power analysis outside of prospective studies are not required, even though they can be calculated, p values are sufficient. Medical readers without a research or statistics background may be confused.

Summary:

- What are the main claims of the paper and how significant are they for the discipline? *This paper claims that there may be association between intraoperative bleeding and myocardial injury. This is novel and important. It also hints at a level of hemoglobin (“transfusion trigger”), for MINS, which is different to normal clinical practice. If this were further substantiated, it would be practice-changing.*
- Are the claims properly placed in the context of the previous literature? Have the authors treated the literature fairly? *There is relatively little prior literature cited, however this is an active area of research and a full exposition would be an article in itself. The conceptualisation of the origin of MINS requires refining as described above.*
- Do the data and analyses fully support the claims? If not, what other evidence is required? *The data available has been correctly analysed and put through statistical tests for unmeasured confounding. However, a significant cause for concern is the different risk profile at baseline of the two groups, which has the potential to heavily confound the paper’s conclusions. This is a fundamental limitation. The multiple primary analyses are confusing and should be reworked: choose one definition of bleeding, and then do secondary analyses for interaction with transfusion +/- other relevant factors.*
- *PLOS ONE* encourages authors to publish detailed protocols and algorithms as supporting information online. Do any particular methods used in the manuscript warrant such treatment? If a protocol is already provided, for example for a randomized controlled trial, are there any important deviations from it? If so, have the authors explained adequately why the deviations occurred? *The authors have provided some statistical analysis detail in supplementary material already.*
- If the paper is considered unsuitable for publication in its present form, does the study itself show sufficient potential that the authors should be encouraged to resubmit a revised version? *This is a good dataset and there is material there for a publishable paper, but it needs substantial reworking.*
- Are original data deposited in appropriate repositories and accession/version numbers provided for genes, proteins, mutants, diseases, etc.? *A weblink is provided to the registration site for the study (I think, my Korean is poor) but I couldn’t find the primary data. This is normal practice for medical research: open availability of databases is rare.*
- Are details of the methodology sufficient to allow the experiments to be reproduced? *The statistical methodology requires clarification as described at length above.*
- Is the manuscript well organized and written clearly enough to be accessible to non-specialists? *The organisation of the manuscript is conventional, and it can be made readable. The subject matter and statistical techniques are relatively specific to medical epidemiology and perioperative medicine however, so I doubt a paper of this type would ever be that accessible to non-specialists realistically.*

Bottom line: this can be made into a paper that contributes to our understanding of risk factors for MINS, but it’s interpretability will be fundamentally limited by it’s unequal baseline characteristics in the event and non-event group.

Yours sincerely,

References:

1.	Investigators VS. Association Between Postoperative Troponin Levels and 30-Day Mortality Among Patients Undergoing Noncardiac Surgery. JAMA. 2012;307(21):2295.

2.	Kristensen SD, Knuuti J, Saraste A, Anker S, Botker HE, Hert SD, et al. 2014 ESC/ESA Guidelines on non-cardiac surgery: cardiovascular assessment and management: The Joint Task Force on non-cardiac surgery: cardiovascular assessment and management of the European Society of Cardiology (ESC) and the European Society of Anaesthesiology (ESA). Eur Heart J. 2014;35(35):2383-431.
