# Supplementary material for: Intraoperative blood loss may be associated with myocardial injury after non-cardiac surgery
Source: PLoS One. 2021 Feb 24;16(2):e0241114. doi: 10.1371/journal.pone.0241114 (PMC7904206; doi:10.1371/journal.pone.0241114)
Supplement: Attachment — Submitted filename: response to reviewers - PONE-D-20-14264R1.docx [file pone.0241114.s008.docx]

**Response to the Reviewers**

**Reviewer #1.**

**Major comments**

**Introduction: our current understanding of MINS is not, as the authors state, principally myocardial oxygen supply/demand imbalance (which more accurately describes Type 2 myocardial infarction according to the 4th universal definition as published by the American College of Cardiology) but is pathophysiologically undefined. Exact causation has not been established but it is likely a heterogenous syndrome of cardiac myocyte necrosis secondary to ischemia, inflammation, immunological stress reaction (mostly through circulating cytokines such as TNF-alpha), and neuroendocrine dysregulation, and as such has a large number of potential clinical correlates. MINS is therefore more accurately a biomarker for increased postoperative morbidity and mortality from a range of sources.**

>> Response: We thank you for your kind comments. Following your recommendation, we changed the Introduction section as below.

“During surgical procedures, massive blood loss can be frequently encountered and can lead to decreased hemoglobin level, and oxygen supply/demand mismatch has been proposed as one of mechanisms for the occurrence of MINS [3].” (Line 78)

**The authors have excluded patients with conditions that cause raised troponin elevation (such as renal impairment, heart failure, sepsis, atrial fibrillation, pulmonary embolism etc.) as these are regarded as artefactual troponin rises. This is can be a legitimate decision, but requires justification. These conditions are often co-morbid with MINS as they share a common pathology (inflammatory response to tissue trauma), so true MINS incidence may be missed. This could be screened for using a sensitivity analysis, as was done for renal impairment and heart failure in the original VISION paper (1), where the MINS concept was first popularised.**

>> Response: We agree with reviewer that other conditions related to troponin elevation should have been more carefully analysed, so we added the sensitivity analysis as below.

**“**For sensitivity analysis, we evaluated whether the observed association was significant in patients with chronic kidney disease and heart failure and after January 2017 when the threshold of hemoglobin for intraoperative transfusion was lowered to 7 g/dL.” (Line 166)

**“**In the sensitivity analysis, this association was consistently found to be significant in patients with chronic kidney disease and heart failure and after lowering the threshold of hemoglobin for intraoperative transfusion in 2017 (S1 Table).” (Line 197)

**“**The observed association between intraoperative bleeding was shown to be significant in most of subgroups with risk factor for MINS.” (Line 287)

**Supplemental table 1.** Sensitivity Analysis of the Observed Association between Significant Bleeding and Myocardial Injury after Noncardiac Surgery

|  | OR (95% CI) | P-value |
| --- | --- | --- |
| Patients with heart failure (n=327) | 4.63 (2.42-9.05) | <0.001 |
| Patients with chronic kidney disease (n=1012) | 1.17 (0.86-1.59) | <0.001 |
| Surgeries after 2017 with the threshold of  hemoglobin level < 7 g/dL for intraoperative transfusion | 2.20 (1.94-2.50) | <0.001 |

OR, odds ratio; CI, confidence interval; RBC, red blood cell

**There is a significant clinical and statistical difference in baseline characteristics between the bleeding and non-bleeding cohorts, with regard to anemia, alcohol use, chronic kidney disease, active cancer, being in ICU, continuous renal replacement therapy, ventilation, surgical risk, emergency operation status and requirement for continuous inotropic support. All of these affect the risk of MINS, and therefore confound the effect of bleeding on the incidence of MINS. This is a major limitation.**

>> Response: We fully agree with the reviewer that there is a large difference in baseline characteristics of the two groups. So, we conducted inverse probability weighting for statistical adjustment for the difference. The result was not changed after mor rigrous statistical adjustment. Addistionally for clarification, we added in the limitation section that it could stll be biased by unmaseured confounders despite statistical adjustment.

” To retain a large sample size and maximize the study power while maintaining a balance in covariates between the two groups, we conducted rigorous adjustment for differences in baseline characteristics of patients using the weighted regression models with the inverse probability weighting (IPW) [19]. According to this technique, weights for patients without significant bleeding were the inverse of the propensity score and weights for patients with significant bleeding were the inverse of 1 – the propensity score.” (Line 160)

“First, this is a single-center, observational study; the possibility of selection bias or unmeasured confounding factors exists. Also, our data may not be generalized to populations in other countries considering ethnic differences in blood management.” (Line 307)

**Table 1. Baseline characteristics according to significant intraoperative bleeding.**

|  | **Entire population** | | | **IPW** | | |
| --- | --- | --- | --- | --- | --- | --- |
|  | **No significant bleeding**  **(N = 13416)** | **Significant bleeding**  **(N = 2510)** | **ASD** | **No significant bleeding**  **(N = 13370.5)** | **Significant bleeding**  **(N = 2644.5)** | **ASD** |
| Male | 8133 (60.6) | 1562 (62.2) | 3.3 | 8145.0 (60.9) | 1658.9 (62.7) | 3.7 |
| Age | 61.7 (±13.5) | 60.0 (±13.5) | 12.4 | 61.5 (±13.6) | 62.2 (±13.3) | 5.0 |
| Preoperative anemia | 4949 (36.9) | 1646 (65.6) | 59.9 | 5497.0 (41.1) | 1064.8 (40.3) | 1.7 |
| Diabetes | 7890 (58.8) | 1748 (69.6) | 22.7 | 8019.3 (60.0) | 1446.4 (54.7) | 10.7 |
| Hypertension | 7290 (54.3) | 1424 (56.7) | 4.8 | 7331.9 (54.8) | 1436.0 (54.3) | 1.1 |
| Current smoking | 1362 (10.2) | 236 (9.4) | 2.5 | 1337.6 (10.0) | 282.8 (10.7) | 2.3 |
| Current alcohol | 2798 (20.9) | 360 (14.3) | 17.2 | 2639.6 (19.7) | 556.3 (21.0) | 3.2 |
| Chronic kidney disease | 680 (5.1) | 332 (13.2) | 28.6 | 850.8 (6.4) | 166.3 (6.3) | 0.3 |
| History of ischemic heart disease | 1913 (14.3) | 365 (14.5) | 0.8 | 1939.1 (14.5) | 395.5 (15.0) | 1.3 |
| History of heart failure | 282 (2.1) | 45 (1.8) | 2.2 | 277.8 (2.1) | 51.3 (1.9) | 1.0 |
| History of stroke | 938 (7.0) | 161 (6.4) | 2.3 | 926.8 (6.9) | 176.3 (6.7) | 1.1 |
| History of arrhythmia | 882 (6.6) | 161 (6.4) | 0.6 | 875.4 (6.5) | 179.4 (6.8) | 0.9 |
| History of heart valve disease | 149 (1.1) | 21 (0.8) | 2.8 | 143.2 (1.1) | 25.9 (1.0) | 0.9 |
| Active cancer | 7422 (55.3) | 1112 (44.3) | 22.2 | 7216.2 (54.0) | 1555.1 (58.8) | 9.8 |
| Preoperative care |  |  |  |  |  |  |
| RBC transfusion | 608 (4.5) | 151 (6.0) | 6.6 | 638.0 (4.8) | 123.2 (4.7) | 0.5 |
| Intensive care unit | 424 (3.2) | 278 (11.1) | 31.2 | 574.7 (4.3) | 111.4 (4.2) | 0.4 |
| ECMO | 0 | 1 (0.0) | 2.8 | 0 | 0.2 (0.0) | 1.1 |
| Continuous renal replacement therapy | 18 (0.1) | 54 (2.2) | 19.1 | 54.7 (0.4) | 11.3 (0.4) | 0.3 |
| Ventilator | 67 (0.5) | 56 (2.2) | 15.0 | 101.2 (0.8) | 18.9 (0.7) | 0.5 |
| Operative variables |  |  |  |  |  |  |
| ESC/ESA surgical high risk | 3997 (29.8) | 1258 (50.1) | 42.4 | 4306.0 (32.2) | 748.8 (28.3) | 8.5 |
| Emergency operation | 1521 (11.3) | 549 (21.9) | 28.6 | 1745.6 (13.1) | 354.4 (13.4) | 1.0 |
| General anesthesia | 13305 (99.2) | 2500 (99.6) | 5.5 | 13267.8 (99.2) | 2610.5 (98.7) | 5.2 |
| Operation duration, hours | 3.94 (±2.14) | 5.57 (±2.86) | 64.4 | 4.20(±2.51 | 4.16±2.20) | 1.7 |
| Continuous infusion of inotropes | 4592 (34.2) | 1278 (50.9) | 34.2 | 4855.3 (36.3) | 798.6 (30.2) | 13.0 |
| Types of surgery |  |  | >0.99 |  |  |  |
| Vascular | 981 (7.3) | 169 (6.7) |  |  |  |  |
| Orthopedic | 667 (5.0) | 146 (5.8) |  |  |  |  |
| Neuro | 3317 (24.7) | 166 (6.6) |  |  |  |  |
| Breast or Endo | 148 (1.1) | 22 (0.9) |  |  |  |  |
| Plastic or Otolaryngeal or Eye | 279 (2.1) | 53 (2.1) |  |  |  |  |
| Transplantation | 300 (2.2) | 768 (30.6) |  |  |  |  |
| Gynecology or Urology | 823 (6.1) | 258 (10.3) |  |  |  |  |
| Gastrointestinal | 4559 (34.0) | 741 (29.5) |  |  |  |  |
| Noncardiac thoracic | 2328 (17.4) | 184 (7.3) |  |  |  |  |
| Others | 14 (0.1) | 3 (0.1) |  |  |  |  |

Data are presented as n (%) or mean (±standard deviation)

IPW, inverse probability weighting; ASD, absolute standardized mean difference; RBC, red blood cell; ECMO, extracorporeal membranous oxygenation; RAAS, renin-angiotensin-aldosterone system; ESC, European Society of Cardiology; ESA, European Society of Anaesthesiology

**The authors state that they have made an estimate of the effect of potential unmeasured confounders (presumably with a sensitivity analysis with a null variable although this is not explicitly stated). This technique requires more explanation, particularly in view of the very disparate and potentially powerfully confounding medical statuses of the two groups in the cohort. Sensitivity analysis is usually used in epidemiology to model the potential effect of a variable which is known or postulated from prior experience or research, but unmeasured in the study at hand. For example, “I think having X increases risk of MINS by OR 2, what effect would it have on my results if it 20% of my MINS group had X? What about 40%? Etc.” This enables you to make a statement such as “Even if X were present in one of my groups with prevalence up to 40%, it would not have changed the result”. How it was used in this study requires more explanation in the text. This technique does not compensate for the confounding caused by the major differences in medical status and operation type mentioned already.**

>> Response: We agree that the statistical method and results of our sensitivity analysis needs more explanation. So, we added as below to the Method and Result sections.

**“**We also estimated the potential impact of unmeasured confounders by calculating the change of OR and CI according to the associations of unmeasured confounders with significant bleeding and MINS with an assumed unmeasured confounder with prevalence of 40% [20].” (Line 168)

“Significant bleeding was associated with MINS, even if the assumed unmeasured confounders were present in one of our groups with prevalence up to 40%. (S2 Table)” (Line 199)

**The authors analyse their data with two different definitions of significant blood loss (including and excluding whether transfusion was required). This is confusing. They could consider picking one definition of bleeding and analysing that as the primary outcome, and then if they wanted to explore the interaction of transfusion they could do that as a secondary analysis (perhaps using matched pairs controlled for hemoglobin drop or some similar technique). Alternatively, they could divide the groups into a two-by-two table (hemoglobin decrease/transfusion) and just make this the primary reported result (as they did in table s5), skipping the intermediate steps. This would be an important result, as transfusion has it’s own risks that may be associated with MINS, and disentangling it from the effect of the bleeding event itself, and anemia, is important.**

>> Response: We agree that presenting two analyses might be confusing for ther readers. Following your recommendation, we only used definition of significant blood loss as our primary analysis. We conducted for group comparison according to bleeding and intraoperative transfusion and the analysis according to the actual decrease of hemoglobin was presented as a supplemental table.

“The patients for this study were divided according to significant intraoperative bleeding. Significant intraoperative bleeding was defined as an absolute value of intraoperative lowest hemoglobin level < 7 g/dL, relative value intraoperative lowest hemoglobin level less than 50% of preoperative measurement, or need for intraoperative packed RBC transfusion [10-13].” (Line 116)

**“**After an adjustment with multivariable analysis, the risk for MINS was found to be significantly increased for the significant bleeding group (35.2% vs. 16.4%; OR, 1.80; 95% CI, 1.61–2.00; *p* < 0.001) (Table 2). The result after IPW adjustment also showed an increased risk for MINS for the significant bleeding group (OR, 1.58; 95% CI, 1.43–1.75; *p* < 0.001) (Table 2).” (Line 189)

**“**Compared with the no hemoglobin decrease without transfusion group, the risk for MINS increased according to both hemoglobin decrease and receipt of RBC transfusion (OR, 2.04; 95% CI, 1.83–2.27; *p <* 0.001 for no hemoglobin decrease with transfusion; OR, 6.13; 95% CI, 4.81–7.82; *p <* 0.001 for hemoglobin decrease without transfusion; and OR, 8.66; 95% CI, 6.77–10.96; *p <* 0.001 for hemoglobin decrease with transfusion) (Table 3). When the patients solely divided according to hemoglobin decrease without considering RBC transfusion, 15,353 (96.4%) patients were in the no hemoglobin decrease group and 573 (3.6%) patients were in the hemoglobin decrease group (S4 Table), the incidence of MINS was substantially increased in the hemoglobin decrease group (58.3% vs. 17.9%; OR, 3.28; 95% CI, 2.70–4.00; *p <* 0.001) (S5 and S6 Tables).” (Line 209)

**Table 2. The incidence of myocardial injury after noncardiac surgery and mortality.**

|  |  |  | **Univariable analysis** | | **Multivariable analysis** | | **IPW analysis** | |
| --- | --- | --- | --- | --- | --- | --- | --- | --- |
|  | **No significant bleeding**  **(N = 13416)** | **Significant bleeding**  **(N = 2510)** | **Unadjusted OR/HR (95% CI)** | ***p* value** | **Adjusted OR/HR (95% CI)** | ***p* value** | **Adjusted OR/HR (95% CI)** | ***p* value** |
| MINS | 2200 (16.4) | 883 (35.2) | 2.77 (2.52-3.04) | < 0.001 | 1.80 (1.61-2.00) | < 0.001 | 1.58 (1.43-1.75) | < 0.001 |
| 30-day mortality | 173 (1.3) | 112 (4.5) | 3.52 (2.77-4.46) | < 0.001 | 2.04 (1.55-2.67) | < 0.001 | 2.51 (1.91-3.28) | < 0.001 |
| Cardiovascular | 50 (0.4) | 28 (1.1) | 3.04 (1.91-4.82) | < 0.001 | 1.92 (1.13-3.27) | 0.02 | 1.90 (1.10-3.29) | < 0.001 |
| Noncardiovascular | 123 (0.9) | 84 (3.3) | 3.71 (2.81-4.90) | < 0.001 | 2.10 (1.53-2.88) | < 0.001 | 2.76 (2.02-3.76) | < 0.001 |

Data are presented as n (%)

MINS was presented with OR, and mortalities were presented as HR

IPW, inverse probability weighting; MINS, myocardial injury after noncardiac surgery; OR, odds ratio; HR, hazard ratio; CI, confidence interval

**Table 3. The incidence of myocardial after noncardiac surgery and mortality according to intraoperative hemoglobin decrease and transfusion.**

|  | **No hemoglobin decrease without transfusion**  **(N = 13416)** | **No hemoglobin decrease with transfusion**  **(N = 1937)** | **Hemoglobin decrease without transfusion**  **(N = 275)** | **Hemoglobin decrease with transfusion**  **(N = 298)** |
| --- | --- | --- | --- | --- |
|  |  |  |  |  |
| MINS, No (%) | 2200 (16.4) | 549 (28.3) | 147 (53.5) | 187 (62.8) |
| Unadjusted OR (95% CI) | 1 [reference] | 2.02 (1.81-2.24) | 5.85 (4.60-7.46) | 8.59 (6.77-10.94) |
| *p* value |  | < 0.001 | < 0.001 | < 0.001 |
| 30-day mortality, No (%) | 173 (1.3) | 61 (3.1) | 26 (9.5) | 25 (8.4) |
| Unadjusted HR (95% CI) |  | 2.46 (1.84-3.30) | 7.75 (5.14-11.71) | 6.76 (4.45-10.29) |
| *p* value |  | < 0.001 | < 0.001 | < 0.001 |
| Cardiovascular mortality, No (%) | 50 (0.4) | 17 (0.9) | 9 (3.3) | 2 (0.7) |
| Unadjusted HR (95% CI) |  | 2.37 (1.37-4.11) | 9.23 (4.54-18.78) | 1.87 (0.45-7.67) |
| *p* value |  | < 0.001 | < 0.001 | 0.39 |
| Non-cardiovascular mortality, No (%) | 123 (0.9) | 44 (2.3) | 17 (6.2) | 23 (7.7) |
| Unadjusted HR (95% CI) |  | 2.50 (1.77-3.52) | 7.14 (4.30-11.86) | 8.76 (5.61-13.67) |
| *p* value |  | < 0.001 | < 0.001 | < 0.001 |

Data are presented as n (%)

MINS was presented with OR, and mortalities were presented as HR

MINS, myocardial injury after noncardiac surgery; OR, odds ratio; HR, hazard ratio; CI, confidence interval

**Supplemental table 4.** Baseline Characteristics According to the Actual Hemoglobin Decrease without Regarding Intraoperative Transfusion

|  | **No hemoglobin decrease** | **Hemoglobin decrease** | ***P* Value** |
| --- | --- | --- | --- |
|  | **(N = 15353)** | **(N = 573)** |  |
| Male | 9387 (61.1) | 308 (53.8) | <0.001 |
| Age | 61.6 (±13.5) | 55.8 (±13.5) | <0.001 |
| Preoperative anemia | 6119 (39.9) | 476 (83.1) | <0.001 |
| Diabetes | 9219 (60.0) | 419 (73.1) | <0.001 |
| Hypertension | 8401 (54.7) | 313 (54.6) | 0.99 |
| Current smoking | 1527 (9.9) | 71 (12.1) | 0.07 |
| Current alcohol | 3063 (20.0) | 95 (16.6) | 0.05 |
| Chronic kidney disease | 907 (5.9) | 105 (18.3) | <0.001 |
| History of ischemic heart disease | 2212 (14.4) | 66 (11.5) | 0.06 |
| History of heart failure | 322 (2.1) | 5 (0.9) | 0.06 |
| History of stroke | 1062 (6.9) | 37 (6.5) | 0.73 |
| History of arrhythmia | 1021 (6.7) | 22 (3.8) | 0.01 |
| History of heart valve disease | 165 (1.1) | 5 (0.9) | 0.8 |
| Active cancer | 8372 (54.5) | 162 (28.3) | <0.001 |
| Preoperative care |  |  |  |
| RBC transfusion | 695 (4.5) | 64 (11.2) | <0.001 |
| Intensive care unit | 570 (3.7) | 132 (23.0) | <0.001 |
| ECMO | 1 (0.0) | 0 | >0.99 |
| Continuous renal replacement therapy | 37 (0.2) | 35 (6.1) | <0.001 |
| Ventilator | 90 (0.6) | 33 (5.8) | <0.001 |
| Operative variables |  |  |  |
| ESC/ESA surgical high risk | 4905 (31.9) | 350 (61.1) | <0.001 |
| Emergency operation | 1854 (12.1) | 216 (37.7) | <0.001 |
| General anesthesia | 15235 (99.2) | 570 (99.5) | 0.68 |
| Operation duration, hours | 4.09 (±2.24) | 6.95 (±3.26) | <0.001 |
| Continuous infusion of inotropes | 5483 (35.7) | 387 (67.5) | <0.001 |
| Types of surgery |  |  |  |
| Vascular | 1090 (7.1) | 31 (5.4) |  |
| Orthopedic | 800 (5.2) | 28 (4.9) |  |
| Neuro | 3449 (22.5) | 36 (6.3) |  |
| Breast or Endo | 165 (1.1) | 5 (0.9) |  |
| Plastic or Otolaryngeal or Eye | 318 (2.1) | 15 (2.6) |  |
| Transplantation | 773 (5.0) | 295 (51.5) |  |
| Gynecology or Urology | 1062 (6.9) | 38 (6.6) |  |
| Gastrointestinal | 5176 (33.7) | 112 (19.5) |  |
| Noncardiac thoracic | 2494 (16.2) | 13 (2.3) |  |
| Others | 26 (0.2) | 0 |  |

Data are presented as n (%) or mean (±standard deviation)

RBC, red blood cell; ECMO, extracorporeal membranous oxygenation; RAAS, renin-angiotensin-aldosterone system; ESC, European Society of Cardiology; ESA, European Society of Anaesthesiology

**Supplemental table 5.** The Incidence of Myocardial Injury after Noncardiac Surgery and Mortality According to the Actual Hemoglobin Decrease without Regarding Intraoperative Transfusion

|  | **No hemoglobin decrease**  **(N = 15353)** | **Hemoglobin decrease**  **(N = 573)** | **Unadjusted OR/HR (95% CI)** | ***p* value** | **Adjusted OR/HR (95% CI)** | ***p* value** |
| --- | --- | --- | --- | --- | --- | --- |
| MINS | 2749 (17.9) | 334 (58.3) | 6.41 (5.40-7.61) | < 0.001 | 3.28 (2.70-4.00) | < 0.001 |
| 30-day mortality | 234 (1.5) | 51 (8.9) | 6.11 (4.52-8.27) | < 0.001 | 2.52 (1.75-3.63) | < 0.001 |
| Cardiovascular | 67 (0.4) | 11 (1.9) | 4.58 (2.42-8.66) | < 0.001 | 2.01 (0.94-4.32) | 0.07 |
| Noncardiovascular | 167 (1.1) | 40 (7.0) | 6.73 (4.76-9.50) | < 0.001 | 2.73 (1.80-4.13) | < 0.001 |

**Minor comments**

**Line 135 – I assume “high risk” here refers to operative mortality risk >5% in table 3 of the ESC/ESA guidelines 2014 (2)? This could be made absolutely clear.**

>> Response: Following your recommendation, we clarified the definition.

“High-risk surgery was defined as procedures with mortality risk >5% according to the 2014 European Society of Cardiology/Anesthesiology guidelines [17].” (Line 131)

**Line 65 and elsewhere – the authors give their main result as “16.4% vs. 35.2%; odds ratio, 1.80; 95% confidence interval, 1.61–2.00; p < 0.001” This should read “35.2% vs 16.4%; odds ratio 1.80; 95% confidence interval 1.61-2.00; p<0.001”. This is true for all the odds ratios in the text.**

>> Response: Following your recommendation, we changed the orders throughout the Abstract and Results sections.

**“**After an adjustment with inverse probability weighting, the incidence of MINS was higher in the significant bleeding group (35.2% vs. 16.4%; odds ratio, 1.58; 95% confidence interval, 1.43–1.75; *p* < 0.001).” (Line 65)

“After an adjustment with multivariable analysis, the risk for MINS was found to be significantly increased for the significant bleeding group (35.2% vs. 16.4%; OR, 1.80; 95% CI, 1.61–2.00; *p* < 0.001) (Table 2).” (Line 189)

“When the patients solely divided according to hemoglobin decrease without considering RBC transfusion, 15,353 (96.4%) patients were in the no hemoglobin decrease group and 573 (3.6%) patients were in the hemoglobin decrease group (S4 Table), the incidence of MINS was substantially increased in the hemoglobin decrease group (58.3% vs. 17.9%; OR, 3.28; 95% CI, 2.70–4.00; *p <* 0.001) (S5 and S6 Tables).” (Line 214)

**While the authors tell us that these are patients having high risk surgery, they do not include a breakdown of the types of surgery performed in the table 1 (Baseline Characteristics). This is important enough to be in the main text, as it affects the applicability of these findings to our individual practice settings. In addition, some surgical populations (vascular surgery in particular) are at very high risk of MINS, and a different incidence between groups may confound the data. This should be moved up into the main text from the supplement (from table S1 into table 1). P values should also be provided.**

>> Response: We agree that this needs to be included in the main table. Following your recommendation, we included the types of surgery in the Table 1. However, including the types of surgery as an adjustment variable would be a double adjustment with the risk of surgery. Moreover, it is true that the risk of MINS is reported to be higher for some types of surgery, but it is also likely that the surgeries with different risks are classified within the same type of surgery. For clarification, we also added that our results could be different according to the types of surgery.

“In In addition, this study contains all types of noncaridac surgery, and the result might be different in particular types of surgery.” (Line 310)

**Line 130 – the exclusion criteria of factors that artefactually increase MINS should be moved up into the methods section for clarity.**

>> Response: Following your recommendation, we moved this section to the Method section.

“Among 3,193 patients with postoperative cTn elevation, 110 patients were diagnosed with non-ischemic cause, and 3083 (19.4%) patients were diagnosed with MINS.” (Line 126)

**General – this dataset spans a relatively long period of time, beginning in 2010, during which our understanding of the hazards of transfusion has evolved and trigger levels of hemoglobin for transfusion have dropped from 10 to 7 g/dl. Were there changes to the author’s institutional policy or consensus in practice on transfusion triggers during this time? Did the authors consider a temporal analysis either on lowest hemoglobin before transfusion, or numbers of transfusions?**

>> Response: We agree that including the change of the institutional protocol into an analysis would be helpful for readers. So, we added as below to the Method, Result, and Discussion sections as a sensitivity analysis.

“**“**For sensitivity analysis, we evaluated whether the observed association was significant in patients with chronic kidney disease and heart failure and after January 2017 when the threshold of hemoglobin for intraoperative transfusion was lowered to 7 g/dL.” (Line 166)

**“**In the sensitivity analysis, this association was consistently found to be significant in patients with chronic kidney disease and heart failure and after lowering the threshold of hemoglobin for intraoperative transfusion in 2017 (S1 Table).” (Line 197)

**“**The observed association between intraoperative bleeding was shown to be significant in most of subgroups with risk factor for MINS.” (Line 287)

**Supplemental table 1.** Sensitivity Analysis of the Observed Association between Significant Bleeding and Myocardial Injury after Noncardiac Surgery

|  | OR (95% CI) | P-value |
| --- | --- | --- |
| Patients with heart failure (n=327) | 4.63 (2.42-9.05) | <0.001 |
| Patients with chronic kidney disease (n=1012) | 1.17 (0.86-1.59) | <0.001 |
| Surgeries after 2017 with the threshold of  hemoglobin level < 7 g/dL for intraoperative transfusion | 2.20 (1.94-2.50) | <0.001 |

OR, odds ratio; CI, confidence interval; RBC, red blood cell

**Details of the power analysis outside of prospective studies are not required, even though they can be calculated, p values are sufficient. Medical readers without a research or statistics background may be confused.**

>> Response: Following your recommendation, we removed the power analysis.

**Reviewer #2.**

**Database/registry study with many limitations, most of which were addressed in the text of the paper. They acknowledged that more studies (more rigorously conducted studies to be specific) need to be conducted to support their conclusions and essentially this study is mostly hypothesis generating.**

**Large population included in study and overall well conducted (given limitations of registry) so does add some controversy to literature that is worth considering (changing transfusion 'triggers' in high risk populations to avoid MINS).**

>> Response: We thank you for your kind comments.

**There were significant differences in baseline characteristics in patients who had blood loss versus those that didn't therefore there were many confounding factors that could influence reasons for these patients to have more MINS besides just blood loss, not sure all of this was accounted for by the statistics or fully explained/justified.**

>> Response: We fully agree with the reviewer that there is a large difference in baseline characteristics of the two groups. So, we conducted inverse probability weighting for statistical adjustment for the difference. The result was not changed after mor rigrous statistical adjustment.

” To retain a large sample size and maximize the study power while maintaining a balance in covariates between the two groups, we conducted rigorous adjustment for differences in baseline characteristics of patients using the weighted regression models with the inverse probability weighting (IPW) [19]. According to this technique, weights for patients without significant bleeding were the inverse of the propensity score and weights for patients with significant bleeding were the inverse of 1 – the propensity score.” (Line 160)

For clarification, we added in the limitation section that it could stll be biased by unmaseured confounders despite statistical adjustment.

“First, this is a single-center, observational study; the possibility of selection bias or unmeasured confounding factors exists. Also, our data may not be generalized to populations in other countries considering ethnic differences in blood management.” (Line 307)

**Table 1. Baseline characteristics according to significant intraoperative bleeding.**

|  | **Entire population** | | | **IPW** | | |
| --- | --- | --- | --- | --- | --- | --- |
|  | **No significant bleeding**  **(N = 13416)** | **Significant bleeding**  **(N = 2510)** | **ASD** | **No significant bleeding**  **(N = 13370.5)** | **Significant bleeding**  **(N = 2644.5)** | **ASD** |
| Male | 8133 (60.6) | 1562 (62.2) | 3.3 | 8145.0 (60.9) | 1658.9 (62.7) | 3.7 |
| Age | 61.7 (±13.5) | 60.0 (±13.5) | 12.4 | 61.5 (±13.6) | 62.2 (±13.3) | 5.0 |
| Preoperative anemia | 4949 (36.9) | 1646 (65.6) | 59.9 | 5497.0 (41.1) | 1064.8 (40.3) | 1.7 |
| Diabetes | 7890 (58.8) | 1748 (69.6) | 22.7 | 8019.3 (60.0) | 1446.4 (54.7) | 10.7 |
| Hypertension | 7290 (54.3) | 1424 (56.7) | 4.8 | 7331.9 (54.8) | 1436.0 (54.3) | 1.1 |
| Current smoking | 1362 (10.2) | 236 (9.4) | 2.5 | 1337.6 (10.0) | 282.8 (10.7) | 2.3 |
| Current alcohol | 2798 (20.9) | 360 (14.3) | 17.2 | 2639.6 (19.7) | 556.3 (21.0) | 3.2 |
| Chronic kidney disease | 680 (5.1) | 332 (13.2) | 28.6 | 850.8 (6.4) | 166.3 (6.3) | 0.3 |
| History of ischemic heart disease | 1913 (14.3) | 365 (14.5) | 0.8 | 1939.1 (14.5) | 395.5 (15.0) | 1.3 |
| History of heart failure | 282 (2.1) | 45 (1.8) | 2.2 | 277.8 (2.1) | 51.3 (1.9) | 1.0 |
| History of stroke | 938 (7.0) | 161 (6.4) | 2.3 | 926.8 (6.9) | 176.3 (6.7) | 1.1 |
| History of arrhythmia | 882 (6.6) | 161 (6.4) | 0.6 | 875.4 (6.5) | 179.4 (6.8) | 0.9 |
| History of heart valve disease | 149 (1.1) | 21 (0.8) | 2.8 | 143.2 (1.1) | 25.9 (1.0) | 0.9 |
| Active cancer | 7422 (55.3) | 1112 (44.3) | 22.2 | 7216.2 (54.0) | 1555.1 (58.8) | 9.8 |
| Preoperative care |  |  |  |  |  |  |
| RBC transfusion | 608 (4.5) | 151 (6.0) | 6.6 | 638.0 (4.8) | 123.2 (4.7) | 0.5 |
| Intensive care unit | 424 (3.2) | 278 (11.1) | 31.2 | 574.7 (4.3) | 111.4 (4.2) | 0.4 |
| ECMO | 0 | 1 (0.0) | 2.8 | 0 | 0.2 (0.0) | 1.1 |
| Continuous renal replacement therapy | 18 (0.1) | 54 (2.2) | 19.1 | 54.7 (0.4) | 11.3 (0.4) | 0.3 |
| Ventilator | 67 (0.5) | 56 (2.2) | 15.0 | 101.2 (0.8) | 18.9 (0.7) | 0.5 |
| Operative variables |  |  |  |  |  |  |
| ESC/ESA surgical high risk | 3997 (29.8) | 1258 (50.1) | 42.4 | 4306.0 (32.2) | 748.8 (28.3) | 8.5 |
| Emergency operation | 1521 (11.3) | 549 (21.9) | 28.6 | 1745.6 (13.1) | 354.4 (13.4) | 1.0 |
| General anesthesia | 13305 (99.2) | 2500 (99.6) | 5.5 | 13267.8 (99.2) | 2610.5 (98.7) | 5.2 |
| Operation duration, hours | 3.94 (±2.14) | 5.57 (±2.86) | 64.4 | 4.20(±2.51 | 4.16±2.20) | 1.7 |
| Continuous infusion of inotropes | 4592 (34.2) | 1278 (50.9) | 34.2 | 4855.3 (36.3) | 798.6 (30.2) | 13.0 |
| Types of surgery |  |  | >0.99 |  |  |  |
| Vascular | 981 (7.3) | 169 (6.7) |  |  |  |  |
| Orthopedic | 667 (5.0) | 146 (5.8) |  |  |  |  |
| Neuro | 3317 (24.7) | 166 (6.6) |  |  |  |  |
| Breast or Endo | 148 (1.1) | 22 (0.9) |  |  |  |  |
| Plastic or Otolaryngeal or Eye | 279 (2.1) | 53 (2.1) |  |  |  |  |
| Transplantation | 300 (2.2) | 768 (30.6) |  |  |  |  |
| Gynecology or Urology | 823 (6.1) | 258 (10.3) |  |  |  |  |
| Gastrointestinal | 4559 (34.0) | 741 (29.5) |  |  |  |  |
| Noncardiac thoracic | 2328 (17.4) | 184 (7.3) |  |  |  |  |
| Others | 14 (0.1) | 3 (0.1) |  |  |  |  |

Data are presented as n (%) or mean (±standard deviation)

IPW, inverse probability weighting; ASD, absolute standardized mean difference; RBC, red blood cell; ECMO, extracorporeal membranous oxygenation; RAAS, renin-angiotensin-aldosterone system; ESC, European Society of Cardiology; ESA, European Society of Anaesthesiology

**Few errors in supplemental tables that need to be corrected - Supplemental Table 1 has a category of surgery "Orthopediatric" since all patients were above age 18 I can only assume this should be 'Orthopedic'. Supplemental Table 3 says "ionotropics" should read 'ionotropes'**

>> Response: These points were clearly our mistake. We changed “Orthopediatric” to “Orthopedic”, and “inotropics” to “inotropes”
