# Supplementary material for: Intraoperative blood loss may be associated with myocardial injury after non-cardiac surgery
Source: PLoS One. 2021 Feb 24;16(2):e0241114. doi: 10.1371/journal.pone.0241114 (PMC7904206; doi:10.1371/journal.pone.0241114)
Supplement: Attachment — Submitted filename: responseletter_20816.docx [file pone.0241114.s009.docx]

**Response to the Editor**

**A. Please address the comments from reviewer 2 and review the manuscript in its entirety to avoid further grammar mistakes.**

>> Response: We thank you for your kind comments. Following your recommendation, we had the whole script proofread by a professional editor.

**B. Please clarify in your response letter the potential overlap between this manuscript and the one submitted to the European Journal of Anaesthesiology on a similar topic. Please delineate the items that are similar and the ones which are unique to this contribution to avoid duplication.**

>> Response: Regarding this, the reviwer and we have exchanged the letters. We explained to the reviewer as below.

“These two studies share the entire cohort, but the enrolled study populations of the two studies differ as well as their conclusions and the clinical implications.

Using the entire cohort, we first analzed the effect of "preoperative anemia' after enrolling all patients with preoeprative hemoglobin level. However, we also felt the need for further analysis on the effect of intraoperative hemoglobin level, because the recent concept of patient blood management covers maintaining an adequate hemoglobin level during pre-, intra, and postoperative periods. Owing to the retrospective nature of our cohort, not every patients with preoperative hemoglobin level had an intraoperartive measurement. So, for intraoperative hemoglobin level, we had to conduct a separate analysis after exculding those without an intraoperative measurement. The study on "preoperative anemia" was conducted among 35,170 patients, and this study on "intraoperative hemoglobin level" exluded 19,2297 patients without intraoperative hemoglobin level and was conducted in 15,926 patients.

These two studies highlight the importance on maintaining an adequate hemoglobin level to decrease MINS, but during the different period by using different study populations.

 I would like to clarify that we had no intention to duplicate or hide our former analysis, but it was an inevitable decision for us to conduct separate analyses for pre- and intraopearive hemoglobin level.”

Now that the reviwer understands that those two studies are distinct. We also mentioned the result of our analysis on preoperative anemia as below.

“Despite various possible causes, the most frequently proposed mechanism for MINS is oxygen supply/demand mismatch, and our previous study also showed that preoperative anemia was associated with MINS [21]. In this follow-up study, we demonstrated that blood loss during surgical procedures could also increase MINS. So, our results on the association between intraoperative hemoglobin decrease and the increased incidence of MINS can be explained by reduced oxygen-carrying capacity compromising the myocardial oxygen supply while simultaneously requiring higher cardiac output to maintain adequate systemic circulation [22].”

**Response to the Reviewers**

**Reviewer #1.**

**1. It has come to our attention that a similar manuscript from your group "Association between preoperative anemia and myocardial injury after noncardiac surgery" by Dr Seung-Hwa Lee is at revision at the European Journal of Anaesthesiology. In oder to assess the distinction between these manuscripts, please provide us with a copy of the current version of your manuscript under review at the European Journal of Anaesthesiology and provide us with a discussion of how this work differs from the work in your current manuscript at PLOS ONE. This information will be used for internal purposes only, and will not be published if your paper is accepted at our journal. Please also let us know if your manuscript is to be published at the European Journal of Anaesthesiology, in which case you would need to include a discussion in this manuscript of how the works differ. Please feel free to email shepp@plos.org with any questions.**

>> Response: We thank you for your kind comments. As we dicussed on e-mails, the results of the analysis on preoperative anemia is mentioned in the script as below with a citation that needs to be confirmed after the publication of the former study.

“Despite various possible causes, the most frequently proposed mechanism for MINS is oxygen supply/demand mismatch, and our previous study also showed that preoperative anemia was associated with MINS [21]. In this follow-up study, we demonstrated that blood loss during surgical procedures could also increase MINS. So, our results on the association between intraoperative hemoglobin decrease and the increased incidence of MINS can be explained by reduced oxygen-carrying capacity compromising the myocardial oxygen supply while simultaneously requiring higher cardiac output to maintain adequate systemic circulation [22].”

When our previous work is ready to be cited , we will add it as reference 21.

**Reviewer #2.**

**Line 55 - should read: which is 'a' severe and common postoperative complication.**

>> Response: We thank you for your kind comments. We changed all the montioned parts as below and had the whole script proofread by professional editor.

**“**This study aimed to evaluate the association between intraoperative blood loss and myocardial injury after non-cardiac surgery (MINS), which is a severe and common postoperative complication.”

**Line 79 - should read: one of 'the' mechanisms**

>> Response: We changed it as below.

**“**Massive blood loss is frequently encountered during surgical procedures and can lead to decreased hemoglobin levels. Oxygen supply/demand mismatch is a proposed as one of the mechanism for MINS [3].”

**Line 193 - does not make sense grammatically (the word interacted), do you mean "the subgroup analysis demonstrated....was 'confounded' by emergency surgery?**

>> Response: We changed it as below.

**“**Subgroup analysis demonstrated that the association between significant bleeding and MINS was confounded by emergency surgery.”

**Line 259 - additional reference (although done in cardiac surgery, would still support your statements) Transfusion, Jan 10, 2008. The influence of baseline hemoglobin on tolerance of anemia in cardiac surgery. Karkouti et. al.**

>> Response: Following your recommendation, we added the reference.

**“**In this study, we selected patients whose intraoperative hemoglobin level decreased below 7 g/dL, which is generally assumed to be the level that most patients can tolerate [10-12,23].”

**Line 213 - should read: When the patients 'were' solely divided....**

>> Response: We changed it as below.

“When the patients were solely divided according to hemoglobin decrease without considering RBC transfusion, 15,353 (96.4%) patients had no hemoglobin decrease whereas 573 (3.6%) patients did have decreased hemoglobin (S4 Table). The incidence of MINS was substantially increased in the decreased hemoglobin group (58.3% vs. 17.9%; OR, 3.28; 95% CI, 2.70–4.00; *p <* 0.001) (S5 and S6 Tables).”

**Line 284 - Although your conclusions are more or less consistent with the idea of restrictive transfusion, they do not completely support the current transfusion thresholds, your results from this study would suggest that the lower trigger limit should perhaps be higher than 7g/dL (although you do mention this point later)**

>> Response: Following your recommendation, we changed it as below.

**“**Despite the current guidelines advocating restrictive use of blood transfusion during surgical procedures, our findings suggest that the hemoglobin threshold for intraoperative transfusion may be higher than 7g/dL in order to prevent MINS [10-12,17].”

**Line 288 - the explanation for lack of correlation of emergency surgery with MINS makes no sense, one would expect that unstable bleeding patients would experience a greater propensity for MINS (since more supply/demand mismatch due to increased work on the heart and increased cardiac output in these situations with an increase in cytokines/inflammatory markers)**

>> Response: This sentence needed more explanation. We agree that unstable bleeding events act as a cause of MINS. What we meant was that these preoperative events might have already caused enough metabolic stress for MINS and diluted the direct effect of intraoperative blood loss. For clarification, we added as below to the script.

**“**This may be because patients requiring emergency surgery include those with preoperative hemodynamic instability or massive bleeding, which could be strongly associated with cTn elevation. These events causing a large fluctuation of hemoglobin levels from the preoperative period might have diluted the effect of intraoperative blood loss.”
